# BCI, an inhibitor of the DUSP1 and DUSP6 dual specificity phosphatases, enhances P2X7 receptor expression in neuroblastoma cells

**DOI:** 10.3389/fcell.2022.1049566

**Published:** 2022-12-15

**Authors:** María Benito-León, Juan Carlos Gil-Redondo, Raquel Perez-Sen, Esmerilda G. Delicado, Felipe Ortega, Rosa Gomez-Villafuertes

**Affiliations:** ^1^ Department of Biochemistry and Molecular Biology, Faculty of Veterinary Medicine, University Complutense of Madrid, Madrid, Spain; ^2^ Instituto Universitario de Investigación en Neuroquímica (IUIN), Madrid, Spain; ^3^ Instituto de Investigación Sanitaria San Carlos (IdISSC), Madrid, Spain; ^4^ Department of Nanobiotechnology, Institute for Biophysics, BOKU University for Natural Resources and Life Sciences, Vienna, Austria

**Keywords:** P2X7 receptor, neuroblastoma, DUSP1, p38 phosphorylation, Sp1 transcription factor, phosphatase inhibitor, BCI

## Abstract

P2X7 receptor (P2RX7) is expressed strongly by most human cancers, including neuroblastoma, where high levels of P2RX7 are correlated with a poor prognosis for patients. Tonic activation of P2X7 receptor favors cell metabolism and angiogenesis, thereby promoting cancer cell proliferation, immunosuppression, and metastasis. Although understanding the mechanisms that control P2X7 receptor levels in neuroblastoma cells could be biologically and clinically relevant, the intracellular signaling pathways involved in this regulation remain poorly understood. Here we show that (E)-2-benzylidene-3-(cyclohexylamino)-2,3-dihydro-1H-inden-1-one (BCI), an allosteric inhibitor of dual specificity phosphatases (DUSP) 1 and 6, enhances the expression of P2X7 receptor in N2a neuroblastoma cells. We found that exposure to BCI induces the phosphorylation of mitogen-activated protein kinases p38 and JNK, while it prevents the phosphorylation of ERK1/2. BCI enhanced dual specificity phosphatase 1 expression, whereas it induced a decrease in the dual specificity phosphatase 6 transcripts, suggesting that BCI-dependent inhibition of dual specificity phosphatase 1 may be responsible for the increase in p38 and JNK phosphorylation. The weaker ERK phosphorylation induced by BCI was reversed by p38 inhibition, indicating that this MAPK is involved in the regulatory loop that dampens ERK activity. The PP2A phosphatase appears to be implicated in the p38-dependent dephosphorylation of ERK1/2. In addition, the PTEN phosphatase inhibition also prevented ERK1/2 dephosphorylation, probably through p38 downregulation. By contrast, inhibition of the p53 nuclear factor decreased ERK phosphorylation, probably enhancing the activity of p38. Finally, the inhibition of either p38 or Sp1-dependent transcription halved the increase in P2X7 receptor expression induced by BCI. Moreover, the combined inhibition of both p38 and Sp1 completely prevented the effect exerted by BCI. Together, our results indicate that dual specificity phosphatase 1 acts as a novel negative regulator of P2X7 receptor expression in neuroblastoma cells due to the downregulation of the p38 pathway.

## 1 Introduction

Extracellular ATP is present at high concentrations within the tumor microenvironment where it is involved in the regulation of cancer growth and progression, and immune response (Di Virgilio and Adinolfi, 2017; Di Virgilio et al., 2018). ATP is the endogenous ligand of purinergic ionotropic (P2X) and metabotropic (P2Y) receptors at the cell membrane. P2X receptors constitute a family of ligand-gated cationic channels with seven mammalian subtypes (P2X1-7). While P2X1-P2X6 receptors are triggered by nanomolar or low micromolar concentrations of ATP, P2RX7 is activated by nearly millimolar concentrations of ATP (North and Surprenant, 2000; North, 2002). Remarkably, P2RX7 exerts dual activity in most cancers depending on its degree of activation. Thus, while overstimulation of the receptor activates a large pore in the membrane that induces tumor cell death and inhibits tumor growth (Di Virgilio et al., 2018), tonic activation of P2RX7 supports the growth, migration, and invasiveness of tumors *in vivo* in several types of cancer (Adinolfi et al., 2012; Qiu et al., 2014; Amoroso et al., 2015; Amoroso et al., 2016). Moreover, P2RX7 activation also promotes cytokine release, angiogenesis, and adaptation to serum deprivation (Adinolfi et al., 2005; Ferrari et al., 2006; Jelassi et al., 2011; Amoroso et al., 2012; Gomez-Villafuertes et al., 2015), thereby favoring cancer cell survival (Gilbert et al., 2019).

P2RX7 is expressed strongly by nearly all human cancers investigated to date, including primary neuroblastoma tumors and cell lines derived from these (Raffaghello et al., 2006). Neuroblastoma is the most common extracranial solid tumor in children, responsible for about 15% of pediatric cancer mortality (Ward et al., 2014). Moreover, about 20% of neuroblastoma tumors disseminate to other regions of the body, with bone, bone marrow and liver the most frequent metastatic niches (Delloye-Bourgeois and Castellani, 2019). The progression of neuroblastoma is frequently associated with high rates of proliferation, even in the absence of trophic support, and several studies have demonstrated that the degree of tumor differentiation influences patient outcome (Brodeur, 2003). Serum withdrawal triggers EGFR-dependent activation of the PI3K/Akt pathway in neuroblastoma cells, inducing the upregulation of *P2rx7* gene expression, which in turn promotes the survival/proliferation of cancer cells in the absence of trophic support (Gomez-Villafuertes et al., 2015). P2RX7 activation ultimately culminates in metabolic reprogramming, favoring the adaptation of neuroblastoma cells to adverse conditions. Thus, P2RX7 stimulation correlates with higher lactate production upon glucose deprivation, the overexpression of several glycolytic enzymes and an increase in the size of intracellular glycogen stores, enabling cells to better adapt to unfavorable ambient conditions (Amoroso et al., 2012). As such, a better understanding of the intracellular signaling pathways that regulate P2RX7 expression in neuroblastoma cells could be biologically and clinically relevant.

Among the intracellular pathways activated by P2RX7 and associated to cell proliferation are the cascades of mitogen-activated protein kinases (MAPKs). MAPKs are Ser/Thr kinases that can phosphorylate a large number of downstream effectors located in the cell nucleus and cytoplasm, but also associated with membranes or the cytoskeleton (Cargnello and Roux, 2011). In mammals MAPKs are grouped into three main families, these containing the extracellular signal-regulated kinases 1 and 2 (ERK1/2), the c-Jun amino-terminal kinases 1 to 3 (JNK1/2/3) and the p38 isoforms (*α*, *β*, *γ*, and *δ*). The activity of the classic ERK1/2 group is mainly triggered by growth factors and mitogens that regulate cell growth, differentiation and development (McKay and Morrison, 2007; Shaul and Seger, 2007). By contrast, the members of the JNK and p38 families are strongly activated by proinflammatory cytokines or in response to environmental stress, and they are preferentially involved in controlling cell proliferation, differentiation, inflammation and apoptosis (Cuenda and Rousseau, 2007; Johnson and Nakamura, 2007; Cuadrado and Nebreda, 2010). The magnitude and duration of MAPKs activation determines their signaling output, which is crucial for numerous biological processes. Indeed, perturbed MAPKs signaling has been involved in the development of many human diseases including various types of cancer (Kim and Choi, 2010). Although MAPK activity can be modulated at different levels, MAPK phosphatases (MKPs) are proving to be the most prominent regulators of the duration and magnitude of MAPKs signaling and activity (Dickinson and Keyse, 2006; Junttila et al., 2008; Patterson et al., 2009). MKPs belong to the superfamily of dual-specificity phosphatases (DUSPs), which act on both Ser/Thr and Tyr residues of their catalytic substrates, being powerful regulators of biological processes. MKPs selectively dephosphorylate MAPKs, driving their catalytic inactivation. Based on sequence homology, subcellular localization, and substrate specificity, the MKPs can be subdivided into three subfamilies. The first includes DUSP1, DUSP2, DUSP4, and DUSP5, all of which are mitogen- and stress-inducible nuclear MKPs. The second group comprises of DUSP6, DUSP7, and DUSP9, which are cytoplasmic ERK-specific MKPs. The final group comprises DUSP8, DUSP10, and DUSP16, which are JNK⁄p38-specific phosphatases that are found in both the cell nucleus and cytoplasm (Caunt and Keyse, 2013).

MKPs, and particularly DUSP1 and DUSP6, have been proposed as cancer biomarkers, since aberrant expression of these phosphatases has been found in a wide variety of human cancers, including neuroblastoma. (Low and Zhang, 2016; Nunes-Xavier et al., 2019). Importantly, the correlation between a particular phosphatase and a tumor phenotype may vary depending on the type of cancer. Thus, MKPs both suppress and promote tumor progression in different cancers, and the same phosphatase may exhibit opposite roles in different tumors (Patterson et al., 2009). In addition, recent advances in the understanding of MKPs biology highlight the potential of these enzymes to be used as targets in cancer therapy, not least since they can be efficiently blocked by small molecule inhibitors (Low and Zhang, 2016; Lazo et al., 2018; Seternes et al., 2019). Using a transgenic zebrafish chemical screen, a biologically active allosteric inhibitor of DUSP1 and DUSP6 was identified, called (E)-2-benzylidene-3-(cyclohexylamino)-2,3-dihydro-1H-inden-1-one (BCI) (Molina et al., 2009). Significantly, purinergic receptors have been revealed as novel regulators of MKPs, which in turn represent additional elements in the nucleotide signaling network (Perez-Sen et al., 2019). Activation of P2RX7 tightly regulates the levels of DUSP6 in cerebellar granule neurons and astrocytes, following a biphasic pattern that involves an early short phase of degradation. This mechanism represents a feedforward mechanism to amplify and prolong ERK1/2 signaling, and it is followed by a second phase in which the expression recovers, exerting a negative feedback mechanism to restore basal ERK1/2 activity (Queipo et al., 2017). However, to date the effect of DUSP on P2RX7 activity has not yet been analyzed. Here, we show that BCI-dependent inhibition of DUSPs produces a potent upregulation of P2RX7 in neuroblastoma cells and that this effect is partially mediated by p38 activation. These results demonstrate that the crosstalk between DUSP proteins and P2RX7 could be relevant in neuroblastoma cell behavior.

## 2 Materials and methods

### 2.1 Antibodies and chemicals

The commercial antibodies used in the study were raised against: P2RX7 (PR-004, Alomone Laboratories); Sp1 (#07-645, Merck Millipore); SAPK/JNK (#9252), anti-phospho-SAPK/JNK (Thr183/Tyr185) (#4668), p38 (#9212), phospho-p38 (Thr180/Tyr182) (#4511) and phospho-Erk1/2 (Thr202/Tyr204) (#4370), all from Cell Signaling; Erk (sc-1647) and DUSP1 (sc-370) from Santa Cruz Biotechnology; DUSP6 (ab76310) from Abcam; GAPDH (G9545) and α-tubulin (T5168) from Sigma-Aldrich. The horseradish peroxidase-conjugated secondary antibodies were from Dako (P0448 and P0260) and the secondary Alexa Fluor^®^ conjugate antibodies (A31572 and A11001) were from Molecular Probes.

The specific inhibitors of MAPKs and other proteins used were: SP600125 (JNK), SB202190 (p38), U0126 (MEK), 666-15 (CREB), SR11302 (AP-1), KJ Pyr 9 (Myc), cyclic Pifithrin *α* (p53) and VO-OH Pic (PTEN), all from Tocris Bioscience; BCI (DUSP1/DUSP6), okadaic acid (PP2A) and BBG (P2RX7) were supplied by Merck-Millipore; mithramycin A (Sp1) and actinomycin D were purchased from Sigma-Aldrich. Other chemicals were supplied by Merck Millipore or Sigma-Aldrich.

### 2.2 Cell culture

Mouse Neuro-2a (N2a, ATCC CCL-131) and human SH-SY5Y (ATCC HTB-11) neuroblastoma cell lines were obtained from ATCC^®^ and grown in DMEM (Gibco), supplemented with 10% FBS (Invitrogen), Glutamax^®^, 100 U/ml penicillin and 100 μg/ml streptomycin, all purchased from Gibco. Cell cultures were grown at 37°C in a humidified atmosphere containing 5% CO_2_. To carry out the experiments, the cells were seeded in complete DMEM medium for 12 h and then transferred to serum-free medium (SFM) for the time periods indicated.

### 2.3 Retrotranscription and real-time quantitative PCR assays

Total RNA was extracted from cells using Speedtools total RNA extraction kit (Biotools), according to the manufacturer’s instructions. After digestion with TURBO DNase (Ambion), the total RNA was quantified with a Nanodrop One spectrophotometer (Thermo Fisher Scientific), and 1 µg of RNA was reversed transcribed using M-MLV reverse transcriptase in the presence of 6 µg of random primers and 350 µM dNTPs (Invitrogen). Quantitative real-time PCR reactions (qPCR) were carried out using LuminoCt^®^ qPCR readymix (Sigma), 5 µl of the cDNA generated, gene-specific primers and TaqMan MGB probes for mouse P2RX7, Sp1, DUSP1, DUSP6, and GAPDH (Applied Biosystems, Madrid, Spain). Fast thermal cycling was performed using a StepOnePlus™ Real-Time PCR System (Applied Biosystems) as follows: pre-denaturation at 95°C for 20 s, followed by 40 cycles each of 95°C for 1 s and 60°C for 20 s. The results were normalized by parallel amplification of the endogenous control GAPDH.

### 2.4 Immunofluorescence

N2a were cultured on coverslips precoated with 0.01 mg/ml poly-L-Lysine (Biochrom) and fixed in 4% paraformaldehyde for 10 min at room temperature (RT). The cells were then washed in PBS and incubated for 1 h at RT in blocking solution (0.3% Triton X-100, 5% goat serum and 10% FBS in PBS). Subsequently, the cells were incubated for 2 h at RT with the primary antibodies against P2RX7 (1:100), DUSP1 (1:100), DUSP6 (1:100), or α-tubulin (1:1,000). After washing twice in PBS containing 2% BSA, the cells were incubated for 1 h at RT with appropriate Alexa Fluor™ conjugated secondary antibodies: donkey anti-rabbit IgG and goat anti-mouse IgG at a 1:500 dilution. Finally, the cells were washed in PBS, the nuclei were counterstained with DAPI, the coverslips were mounted with Aqua Poly/Mount reagent (Polysciences) and confocal images were acquired on a TCS SPE microscope with a 63× Apochromat NA = 1.3 oil objective lens (Leica Microsystems) or with an Eclipse TE2000-E microscope with a 20x Nikon Fluor NA = .45 air objective lens (Nikon). Images were quantified using the ImageJ free software.

### 2.5 MTT cell viability assay

N2a were cultured overnight in complete DMEM and on the following day, the cells were transferred to SFM containing either 5 µM or 10 µM BCI. After 24 h, the metabolic activity was quantified using a MTT assay as follows. The culture medium was replaced with PBS containing the tetrazolium salt 3-(4,5-dimethylthiazol-2-yl)-2,5-diphenyltetrazolium bromide (0.5 mg/ml, Sigma) and the cells were maintained for 2 h in a humidified atmosphere containing 5% CO_2_ at 37°C. Subsequently, an equal volume of solubilization buffer was added (10% Triton X-100 plus 0.1 N HCl in anhydrous isopropanol) and after mixing smoothly for 30 min at RT using an orbital shaker, the cell extracts were finally collected, and the absorbance was quantified at 570 nm. The values were normalized to those obtained from untreated cells, set as 100% metabolic activity.

### 2.6 Calcein/EthD-1 cell viability assay

N2a cells were cultured overnight in complete DMEM and then transferred on the following day to SFM containing either 5 µM or 10 µM BCI. After 24 h, cell viability was analyzed with the LIVE/DEAD^®^ Viability/Cytotoxicity Assay (Invitrogen), replacing the culture medium with PBS containing live-cell staining dye (2 μM calcein-AM) and dead-cell staining dye (2 μM ethidium homodimer-1). Then, cells were incubated for 30 min in a humidified atmosphere containing 5% CO_2_ at 37°C, and fluorescence images were acquired on an Eclipse TE2000-E microscope with a 20x Nikon Fluor NA = .45 air objective lens (Nikon).

### 2.7 Phospho-kinase array

To estimate the relative protein phosphorylation, N2a cells cultured overnight in complete DMEM were transferred to SFM for 30 min and then treated for 1 h with BCI (10 μM) or with the vehicle alone. Cell extracts were prepared using the Human Phospho-Kinase Array Kit (R&D Systems, no. ARY003B) following the manufacturer’s instructions. Although this array was initially developed to identify human phosphoproteins, excellent cross-reactivity with rodent proteins has been demonstrated (Zalfa et al., 2019). After washing the cells with PBS, the diluted cleared cell lysates (400 μg) were incubated overnight with the Phospho-Kinase array, which uses phospho-specific antibodies spotted in duplicate on nitrocellulose membranes. Following multiple washing to remove any unbound protein, the array was incubated with a cocktail of biotinylated antibodies to capture spots corresponding to the phosphorylated proteins using streptavidin–horseradish peroxidase (HRP) and chemiluminescent detection reagents for signal detection. Finally, the signal density was measured with the ImageQuant LAS 500 chemiluminescence imaging system (Amersham GE) and the spot pixel density was analyzed with the ImageJ free software.

### 2.8 Immunoblot assay

N2a cells were lysed for 1 h at 4°C in lysis buffer containing 50 mM Tris/HCl, 150 mM NaCl, 1% Nonidet P40 (pH 7.4), Complete™ Protease Inhibitor Cocktail Tablets (Roche Diagnostics), 1 mM Sodium Orthovanadate (Sigma) and 1.5 µM Okadaic Acid (Calbiochem). The supernatant was harvested by centrifugation, quantified, and equal amounts of protein extract (20 μg) were resolved by 10% Tris-Glycine SDS-PAGE and the proteins transferred to nitrocellulose membranes (Amersham GE). The membranes were then blocked for 1 h at RT with PBS + 0.1% Tween-20 (PBST) buffer containing either 5% skimmed milk or 3% BSA depending on the primary antibody used. The membranes were probed overnight at 4°C with primary antibodies against P2RX7 (1:1,000, 70 kDa), Sp1 (1:1,000, 100 kDa), JNK (1:1,000, 46–54 kDa), phospho-JNK (1:1,000, 46–54 kDa), p38 (1:1,000, 43 kDa), phospho-p38 (1:1,000, 43 kDa), Erk (1:1,000, 42 kDa), phospho-Erk1/2 (1:2,000, 44–42 kDa), and GAPDH (1:10,000, 37 kDa). The next day the membrane was washed twice with PBST and antibody binding was detected with HRP-conjugated secondary antibodies (goat anti-rabbit and rabbit anti-mouse diluted 1:1,000). Protein bands were visualized with an ECL HRP Chemiluminescent Substrate (Perkin Elmer) on an ImageQuant LAS 500 Model and densitometric analysis was performed with ImageQuant software (Amersham GE) using GAPDH as an internal reference.

### 2.9 Statistics

All the data are shown as the mean ± standard error of the mean (SEM) of a minimum of three independent experiments performed in duplicate or triplicate. The data were analyzed using unpaired *t*-test for two-group comparisons or one way-ANOVA with the Dunnett’s *post hoc* test. For multiple comparisons, one-way ANOVA analyses were corrected with Sidak’s *post hoc* test (Graph Pad Prism 6, Graph Pad Software Inc.). A value of *p* ≤ .05 was considered statistically significant.

## 3 Results

### 3.1 DUPS1/DUSP6 inhibition by BCI strongly enhances *P2rx7* gene expression in neuroblastoma cells

**FIGURE 1:**
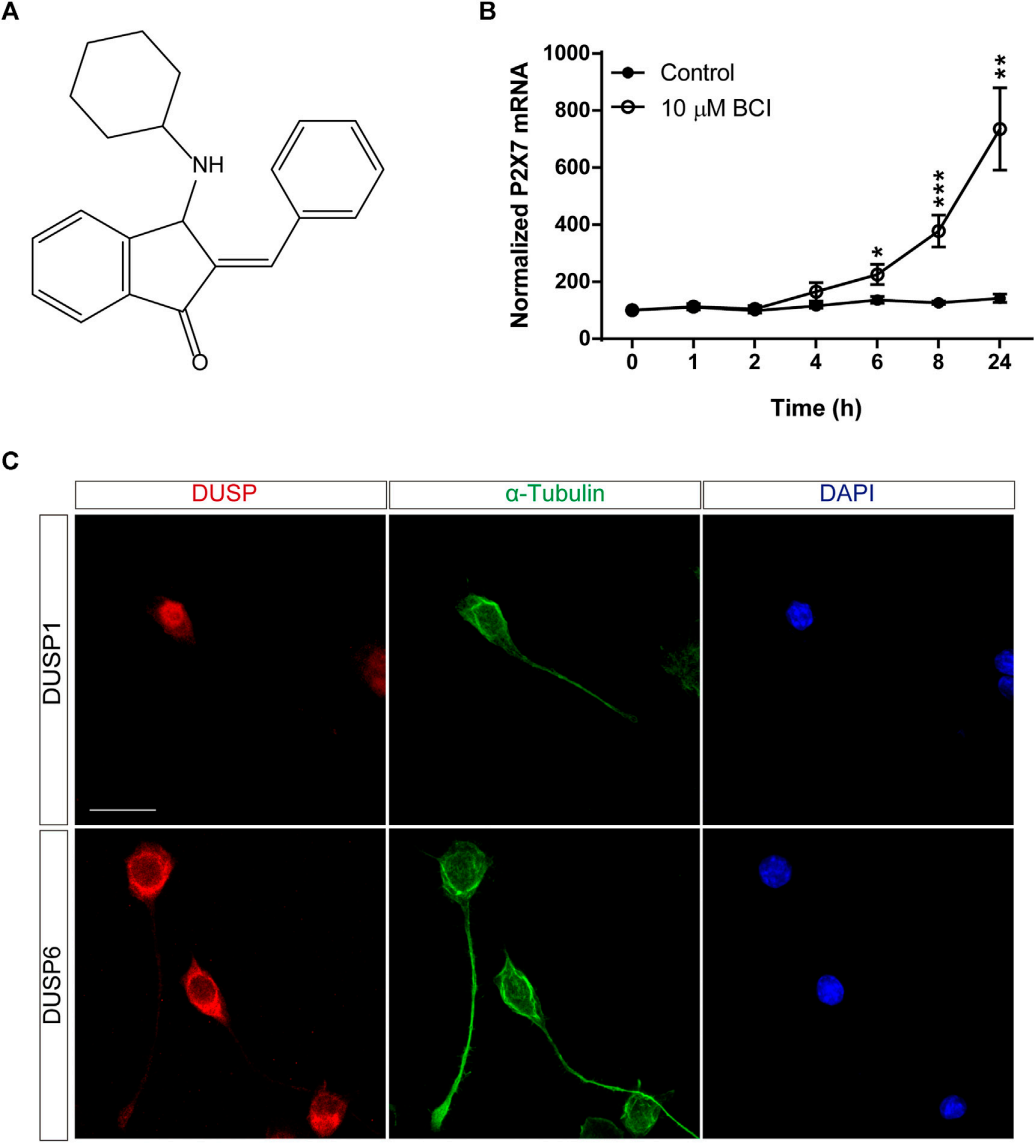
BCI upregulates P2RX7 expression in neuroblastoma cells. **(A)** Chemical structure of BCI. **(B)** N2a cells were incubated in SFM for 1, 2, 4, 6, 8 or 24 h in the presence of BCI (10 μM) or in its absence (control). Total RNA was then extracted from the cells and quantified. The data were normalized to the P2RX7 transcript levels in cells cultured in complete medium, set as 100% (time = 0). The results are the mean ± SEM of five independent experiments performed in triplicate. *p ≤ .05, **p ≤ .01, ***p ≤ .001 vs. control. **(C)** N2a cells cultured for 48 h in SFM, and immunostained for either DUSP1 (upper panel, red) or DUSP6 (lower panel, red) and α-tubulin (green). The nuclei were counterstained with DAPI (blue). Scale bar = 25 μm.

BCI is a small-molecule that acts as a dual-inhibitor of DUSP1 and DUSP6, exerting anti-tumorigenic activity in a wide range of cancer models (Figure 1A) (Kaltenmeier et al., 2017; Wu et al., 2018; James et al., 2019; Ramkissoon et al., 2019; Duan et al., 2021; Nair et al., 2021; Singh et al., 2022). In SH-SY5Y human neuroblastoma cells, BCI elicits a dose-dependent decrease in ERK activity and reduces the viability of these cells (Mendell and MacLusky, 2019). The effect of BCI on P2RX7 expression in N2a cells was analyzed over time in serum-free medium (SFM). Exposure to BCI (10 µM) for 1, 2, 4, 6, 8 or 24 h (time 0 are cells maintained exclusively in complete DMEM medium containing 10% FBS) induced a time-dependent increase in *P2rx7* gene expression, reaching transcript levels up to 8-fold those in the control cells exposed to the vehicle alone (Figure 1B). BCI also enhanced P2RX7 transcripts in N2a cells cultured in complete medium (see Supplementary Figure S1). Moreover, it was confirmed that N2a cells expressed both DUSP1 and DUSP6 phosphatases by immunofluorescence, revealing an intense nuclear distribution of DUSP1 and DUSP6 in the cytosol (Figure 1C).

**FIGURE 2:**
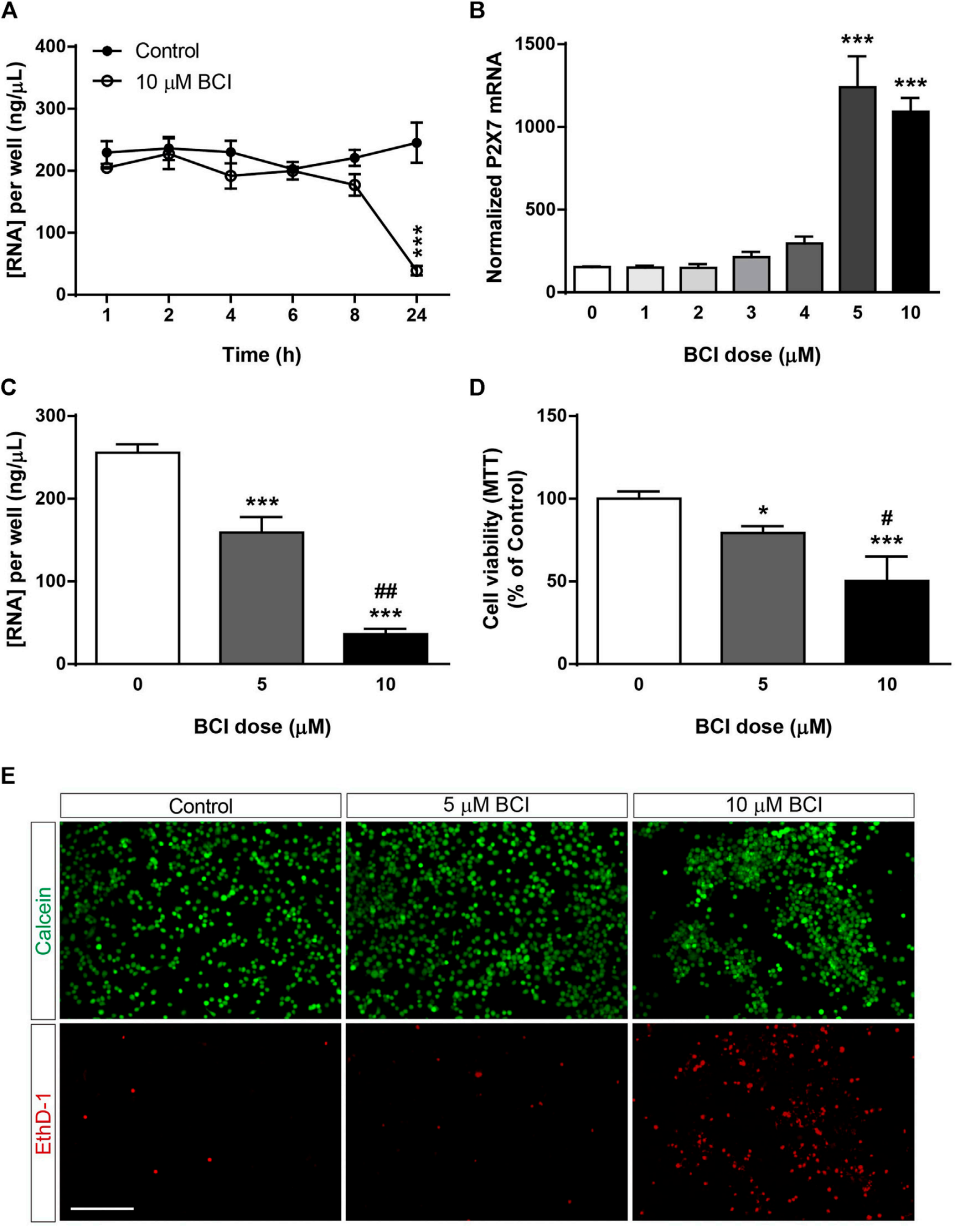
Dose-dependent cytotoxicity of BCI in neuroblastoma cells. **(A)** N2a cells were incubated in SFM for 1, 2, 4, 6, 8 or 24 h in presence of BCI (10 μM) or in its absence (control). Total RNA was then extracted from the cells and quantified. The results are the mean ± SEM of five independent experiments performed in triplicate. ***p ≤ .001 vs. control **(B)** N2a cells were incubated for 24 h in SFM in the presence or absence of increasing concentrations of BCI (ranging from 1 to 10 μM). Total RNA was extracted from the cells, and 1 µg was reversed transcribed and quantified by qPCR, using GADPH as a housekeeping gene and normalizing the P2RX7 expression to that in untreated cells (dose = 0, set as 100%). The results are the mean ± SEM of four independent experiments performed in triplicate. ***p ≤ .001 vs. untreated cells. **(C)** N2a cells were cultured for 24 h in SFM in the presence or absence of either 5 μM or 10 μM BCI. Total RNA was extracted and quantified, showing the results as the mean ± SEM of six independent experiments performed in triplicate. ***p ≤ .001 vs. control; ^##^ *p* ≤ .01 vs. 5 μM BCI. **(D)** MTT assay of N2a cells cultured for 24 h in SFM in the presence or absence of either 5 μM or 10 μM BCI. The values were normalized to those obtained from untreated cells (dose = 0, set as 100%). The results are the mean ± SEM of four independent experiments performed in triplicate. *p ≤ .05, ***p ≤ .001 vs. untreated cells; ^##^ *p* ≤ .01 vs. 5 μM BCI. **(E)** LIVE/DEAD^®^ Viability/Cytotoxicity Assay of N2a cells cultured for 24 h in SFM in the presence or absence of either 5 μM or 10 μM BCI. Viable cells were loaded with calcein (green) whereas the nuclei of dead cells are stained with EthD-1 (red). Scale bar = 200 μm.

Previous studies demonstrated that BCI decreased neuroblastoma cell viability in a dose-dependent and time-dependent manner (Mendell and MacLusky, 2019), although its cytotoxicity appears to be at least partially independent of DUSP1/6 activity (Thompson et al., 2022). The potential toxicity of BCI was evaluated by analyzing the total RNA isolated from N2a cells after they were transferred to SFM and treated with BCI (10 µM) for 1, 2, 4, 6, 8 or 24 h. The total RNA levels remained constant over short time periods relative to control cells, although they decreased significantly after 24 h exposure to BCI, indicating that this DUSP inhibitor produces cytotoxicity after longer exposures (Figure 2A). A dose-response experiment was performed to determine a sub-toxic yet effective concentration of BCI capable of increasing P2RX7 expression in which N2a cells were transferred to SFM and treated for 24 h with doses of BCI ranging from 1 to 10 µM. BCI enhanced P2RX7 transcripts to similar levels when administered at 5 and 10 μM, whereas lower concentrations of BCI were ineffective (Figure 2B). Remarkably, 5 µM BCI was significantly less cytotoxic (Figure 2C), as revealed in MTT and calcein/EthD-1 cell viability assays (Figures 2D,E, respectively). Consequently, 5 μM was the concentration of BCI used in the subsequent long-term experiments (≥24 h).

**FIGURE 3:**
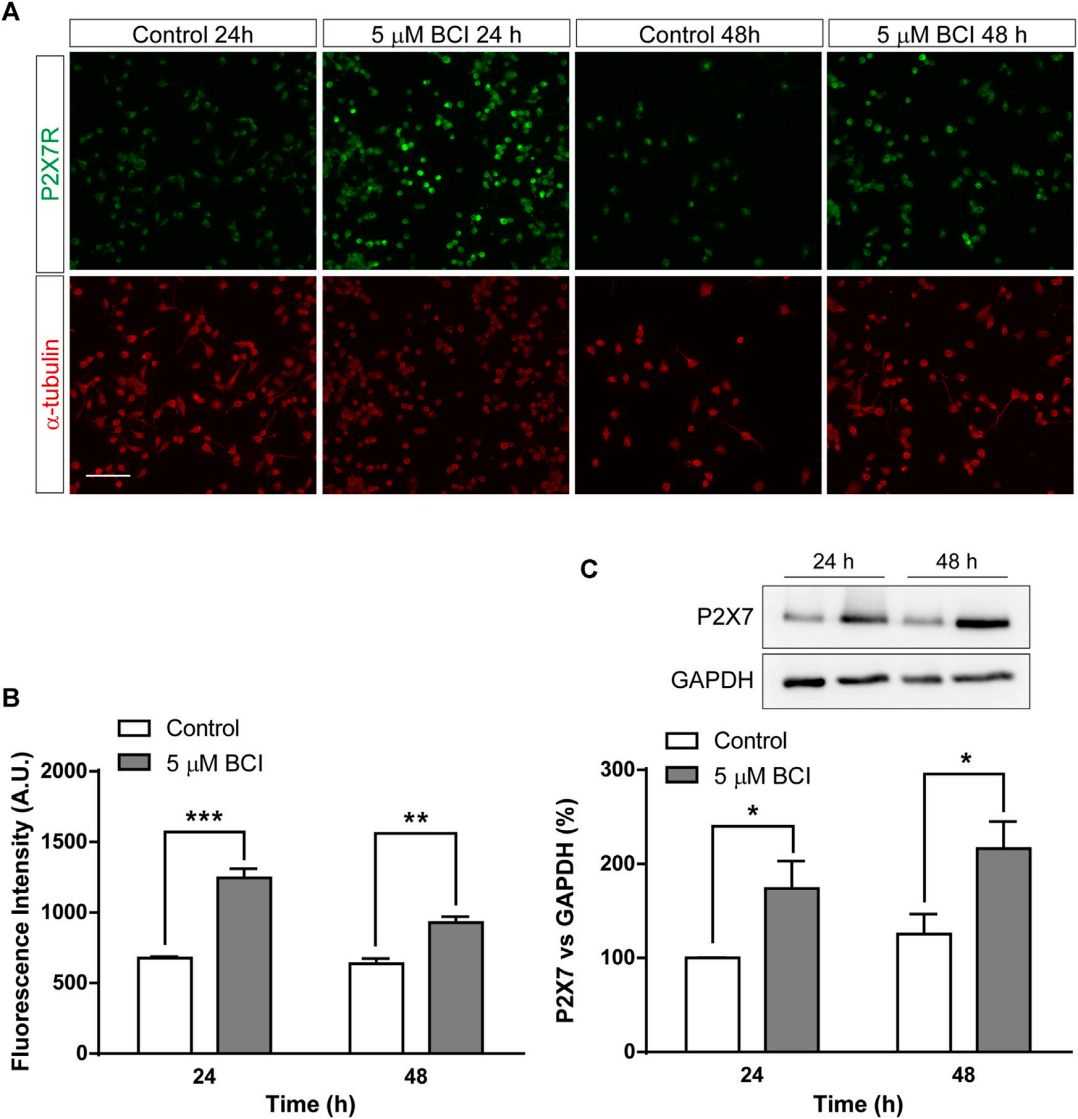
BCI increases the amount of P2RX7 protein in neuroblastoma cells. **(A)** Immunostaining of P2RX7 (green) and α-tubulin (red) in N2a cells cultured in SFM for 24 or 48 h in the presence or absence (control) of BCI (5 μM). Scale bar = 100 μm. **(B)** The histogram represents the quantification of fluorescence intensity of P2RX7-positive cells cultured in SFM for 24 or 48 h in presence or absence of BCI (5 μM). The values were normalized to those obtained in control cells and the results are the mean ± SEM of four independent experiments performed in duplicate. **p ≤ .01, ***p ≤ .001 vs. control. **(C)** Immunoblot depicting the presence of endogenous P2RX7R in cell lysates from N2a cells cultured in SFM for 24 or 48 h in the presence or absence of BCI (5 μM). Western blots of the cell lysates were probed with antibodies against P2RX7R (intracellular epitope). GAPDH was used as internal loading control. The histogram shows P2RX7 protein in control and BCI-treated cells at the time points indicated, obtained by densitometry and normalized to GAPDH. The values represent the mean ± SEM of four independent experiments in duplicate. *p ≤ .05, vs. control.

Since the upregulation of gene expression is not necessarily correlated with a comparable increase in protein, mainly due to the distinct post-transcriptional regulatory mechanisms, we analyzed the effect of BCI on the P2RX7 protein. N2a cells in SFM were treated with BCI (5 µM) for 24 h or 48 h, or with the vehicle alone (control), and the levels of P2RX7 were quantified by immunofluorescence (Figures 3A,B) and immunoblot (Figure 3C). Both these studies demonstrated that a 24 h exposure to BCI significantly enhanced the levels of P2RX7, suggesting that a DUSP1/6-dependent mechanism may be involved in the upregulation of this receptor in neuroblastoma cells. Furthermore, BCI also increased P2X7 transcripts in SHSY5Y human neuroblastoma cells, although to a lesser extent that in N2a cells (see Supplementary Figure S2).

### 3.2 BCI alters the profile of protein phosphorylation in neuroblastoma cells

**FIGURE 4:**
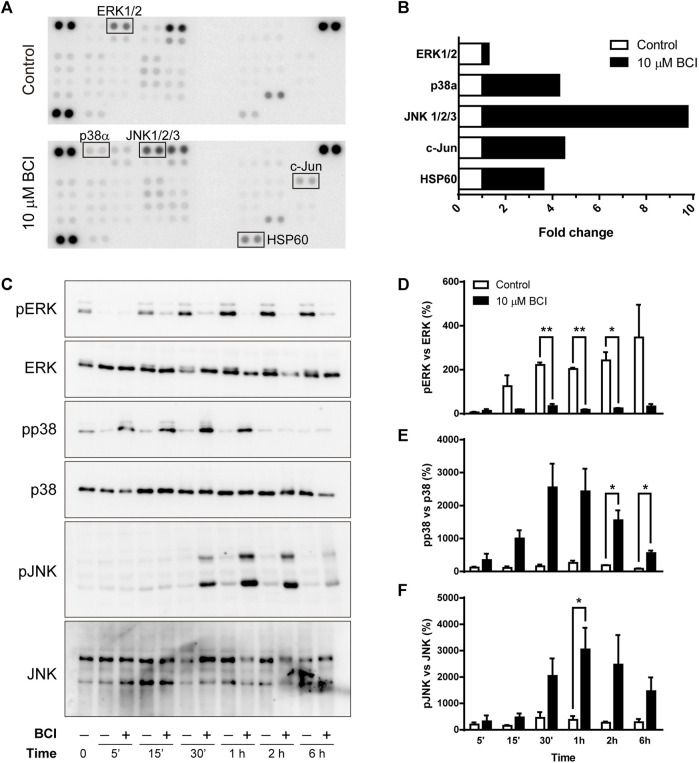
BCI affects the phosphorylation of MAPKs in neuroblastoma cells. **(A)** N2a cells were incubated in SFM for 1 h in the presence or absence (control) of BCI (10 μM), and protein phosphorylation was analyzed with a Phospho-Kinase array assay. Phospho-epitopes that changed at least 2X following BCI treatment are shown in boxes. **(B)** Densitometry analysis of the array shown in panel A indicating the fold-increase of each phosphor-epitope. **(C)** N2a cells were incubated in SFM for 5 min, 15 min, 30 min, 1 h, 2 h or 6 h in presence or absence of BCI (10 μM), with the cells cultured in complete medium shown as time 0. The cell extracts were resolved in immunoblots that were probed to detect the phospho- and total ERK, p38 and JNK MAPKs. The histograms represent phospho-ERK1/2 **(D)**, phospho-p38 **(E)** and phospho-JNK **(F)** protein in control and BCI-treated cells at the time points indicated and they were obtained by densitometry and normalized to the total ERK, p38 and JNK, respectively. Phospho-protein levels where normalized to those found in cells cultured in complete medium, set as 0%. The values represent the mean ± SEM of three independent experiments performed in duplicate. *p ≤ .05, **p ≤ .01 vs. control.

To shed light on the intracellular signaling coupled to the effect of BCI, we examined cell-signaling in an unbiased manner using an array platform that detects multiple protein phosphorylation events (Zalfa et al., 2019). N2a cells were transferred to SFM and treated for 1 h with either BCI (10 µM) or the vehicle (control), and the protein extracts were then analyzed in the Phospho-Kinase array (Figure 4A) to identify those phospho-protein epitopes that were modified at least 2-fold by BCI treatment. The major stress-inducible MAPKs, p38 and JNK were all phosphorylated when cells were exposed to BCI, together with the chaperonin HSP60 and the JNK substrate c-Jun. Conversely, the phosphorylation of ERK1/2 was significantly dampened by BCI relative to control cells (Figure 4B).

As the array analysis was carried out only once, the phosphorylation state of MAPKs was validated in conventional Western blots (Figure 4C). As such, N2a cells in SFM were exposed to BCI (10 µM) for 5 min, 15 min, 30 min, 1 h, 2 h or 6 h, and protein extracts were then examined in immunoblots. The results obtained corroborate the weaker phosphorylation of ERK1/2 (Figure 4D), while both p38 and JNK were phosphorylated transiently in neuroblastoma cells exposed to BCI (Figures 4E,F, respectively). Maximal phosphorylation of both p38 and JNK was observed 1–2 h after treatment, whereas ERK1/2 phosphorylation induced by serum deprivation in control cells was fully abrogated by BCI at all the time points analyzed. Since DUSP6 is an ERK-specific phosphatase, while DUSP1 preferentially dephosphorylates stress-inducible MAPKs, the results obtained suggest that BCI-dependent inhibition of DUSP1 may be responsible for the increase of p38 and JNK phosphorylation.

### 3.3 BCI-dependent activation of p38 prevents ERK phosphorylation in neuroblastoma cells

**FIGURE 5:**
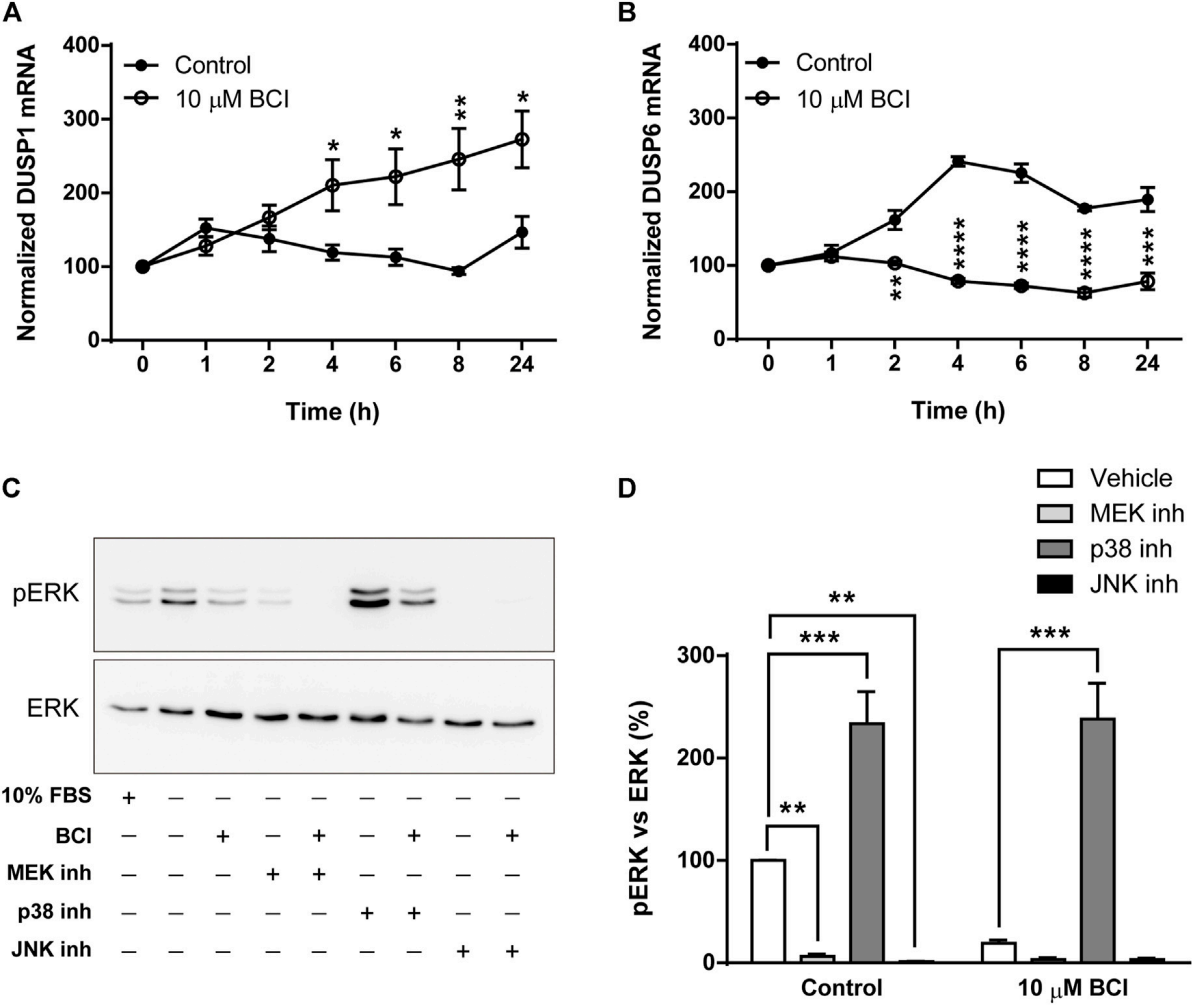
Inhibition of p38 prevents dephosphorylation of ERK1/2 by BCI in neuroblastoma cells. N2a cells were incubated in SFM for 1, 2, 4, 6, 8 or 24 h in the presence or absence (control) of BCI (10 μM). Total RNA was extracted from the cells and quantified. The expression of DUSP1 **(A)** and DUSP6 **(B)** mRNAs was determined and normalized to the DUSP1 or DUSP6 transcript levels in cells cultured in complete medium (time = 0, set as 100%). The results are shown as the mean ± SEM of five independent experiments performed in triplicate. *p ≤ .05, **p ≤ .01, ***p ≤ .001 vs. control. **(C)** N2a cells cultured in SFM were treated with inhibitors of MEK1/2 (10 μM, U0126), p38 (10 μM, SB202190) or JNK (10 μM, SP600125) for 10 min before the addition of BCI (10 µM) or vehicle alone (control) for 1 h. Untreated cells cultured in SFM or complete medium (10% FBS) were also analyzed. The cell extracts were assayed in immunoblots to detect phospho-ERK1/2 and total ERK. **(D)** The histogram shows the phospho-ERK1/2 protein levels obtained by densitometry and normalization to the total ERK. The phospho-protein levels where normalized to those on untreated cells cultured in SFM, set as 100%. The values represent the mean ± SEM of five independent experiments performed in duplicate. **p ≤ .01, ***p ≤ .001 vs. vehicle.

As mentioned previously, BCI is an allosteric inhibitor of DUSP1/6 phosphatases that mainly control the activity of MAPKs through the dephosphorylation of their activation residues. When the DUSP1 and DUSP6 transcripts in N2a cells exposed to BCI (10 µM) for 1, 2, 4, 6, 8 or 24 h in SFM were evaluated, BCI provoked a significant increase in the DUSP1 transcripts relative to the cells exposed to the vehicle alone. Moreover, BCI fully impaired the upregulation of DUSP6 expression induced by serum deprivation in control cells (Figures 5A,B, respectively). Hence, BCI-dependent inhibition of DUSP1 appears to be involved in the phosphorylation of p38 and JNK. Thus, we asked how BCI might block ERK phosphorylation in these cells? One feasible explanation would be the existence of a negative feedback loop between stress-inducible MAPKs and ERK, which was tested by exposing N2a cells in SFM to inhibitors of MEK1/2 (U0126, 10 µM), p38 (SB202190, 10 µM) or JNK (SP600125, 10 µM) for 10 min before incubating them with BCI (10 µM) for 1 h. The proteins isolated from these cells were analyzed by immunoblot to assess the phosphorylation state of ERK. Phospho-ERK1/2 was also quantified in N2a cells cultured in complete serum-containing medium (10% FBS) and we confirmed that the enhanced phosphorylation of ERK upon serum deprivation was prevented by BCI treatment (Figures 5C,D). As expected, inhibiting MEK1/2 abolished ERK phosphorylation in the presence or absence of BCI. Remarkably, the blockade of ERK phosphorylation exerted by BCI was prevented by SB202190 and indeed, the inhibition of p38 significantly increased ERK phosphorylation in control cells, indicating that p38 signaling is involved in the inhibition of the ERK pathway. Conversely, JNK inhibition fully blocked the phosphorylation of ERK, even in control cells not exposed to BCI.

**FIGURE 6:**
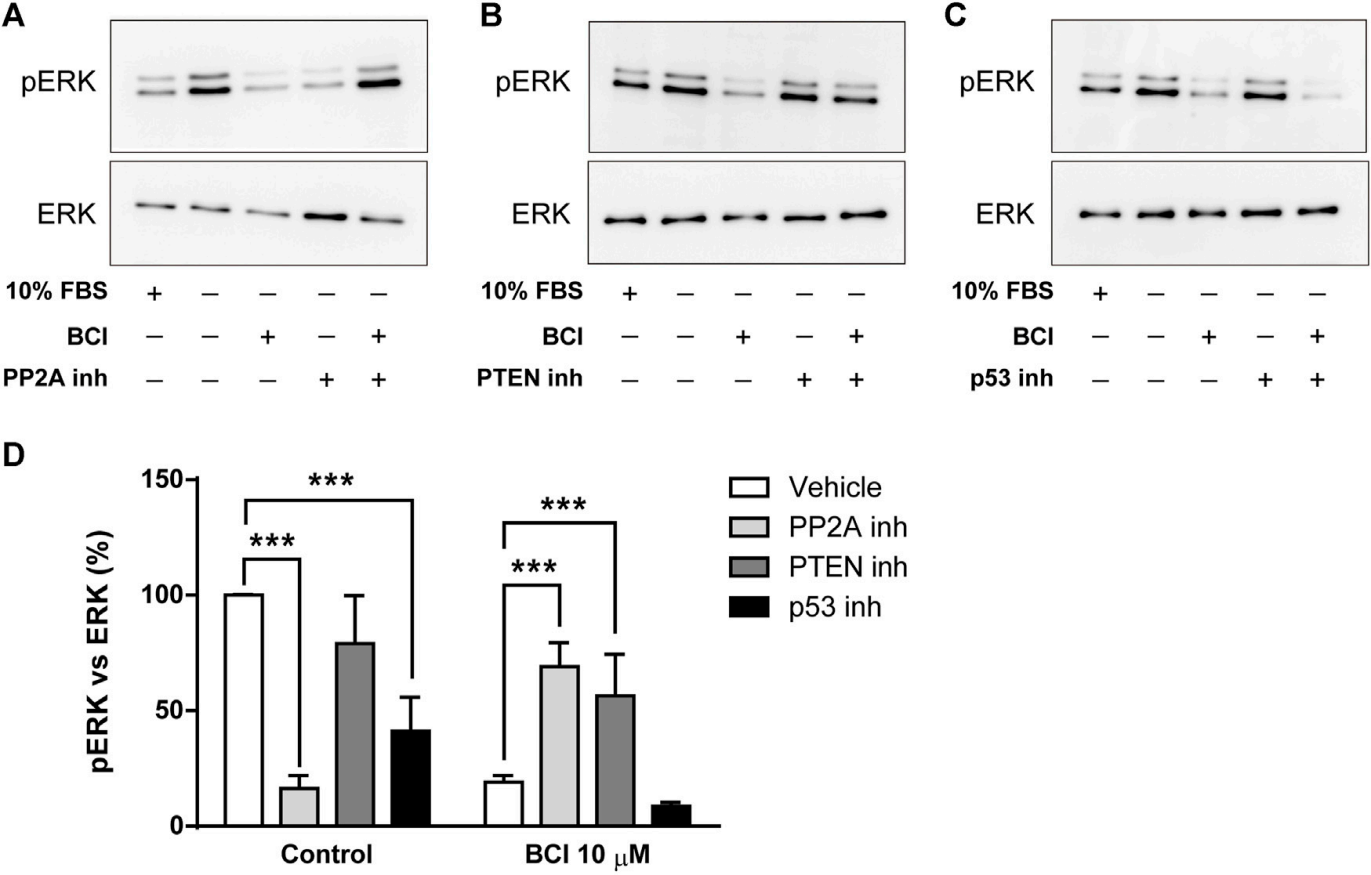
The inhibition of PP2A or PTEN prevents the dephosphorylation of ERK1/2 exerted by BCI in neuroblastoma cells. N2a cells cultured in SFM were treated with inhibitors of **(A)** PP2A (1 μM, okadaic acid), **(B)** PTEN (500 nM, VO-OH pic) or **(C)** p53 (20 μM, cyclic pifithrin-α) for 10 min before the addition for 1 h of either BCI (10 µM) or the vehicle alone (control). Untreated cells cultured either in SFM or complete medium (10% FBS) were also analyzed. All the cell extracts were analyzed in immunoblots to detect phospho-ERK1/2 and total ERK. **(D)** The histogram shows phospho-ERK1/2 protein levels obtained by densitometry and normalized to the total ERK. The phospho-protein levels where normalized to the levels in untreated cells cultured in SFM (set as 100%). The values represent the mean ± SEM of four independent experiments performed in duplicate. ***p ≤ .001 vs. vehicle.

The dephosphorylation of ERK induced by BCI in N2a cells must involve the participation of protein phosphatases other than DUSP1/6 and several studies reported that p38 inhibits ERK activity by enhancing MEK1/2 dephosphorylation through protein phosphatase PP2A (Westermarck et al., 2001; Li et al., 2003; Liu and Hofmann, 2004; Grethe and Porn-Ares, 2006; Junttila et al., 2008). Moreover, the phosphatase and tensin homolog deleted on Chromosome 10 (PTEN) is a tumor suppressor that not only negatively regulates the oncogenic PI3K/Akt pathway to suppress cancer development but also, non-canonical pathways like those involving ERK1/2 (Bouali et al., 2009; Chetram et al., 2011). Thus, N2a cells in SFM and treated with the inhibitors of PP2A (okadaic acid, 1 µM) or PTEN (VO-OH pic, 500 nM) for 10 time before the addition of BCI (10 µM) for 1 h, after which the phosphorylation state of ERK was evaluated by Western blots. Similarly, phospho-ERK1/2 was also quantified in N2a cells cultured in complete serum-containing medium (10% FBS). Strikingly, the inhibition of PP2A reduced the phospho-ERK in control cells but it enhanced ERK phosphorylation in cells exposed to BCI (Figures 6A,D). Moreover, the inhibition of PTEN also increased ERK phosphorylation in BCI-treated cells, although in this case without affecting phospho-ERK levels in control cells (Figures 6B,D). We also analyzed the participation of p53 in the regulation of ERK activity. The transcription factor p53 is a well-known downstream target of p38 that mediates a negative feedback regulation of p38 signaling (Takekawa et al., 2000). There was a significant dampening of ERK phosphorylation in control N2a cells exposed to the p53 inhibitor, cyclic pifithrin-α (20 µM), with no alterations to the phospho-ERK levels in cells treated with BCI (Figures 6C,D). Together these results suggest that the PP2A and PTEN phosphatases appear to be involved in the negative regulation of ERK activity mediated by p38 in N2a cells treated with BCI.

### 3.4 BCI-dependent upregulation of *P2rx7* gene expression is partially reversed by p38 inhibition

**FIGURE 7:**
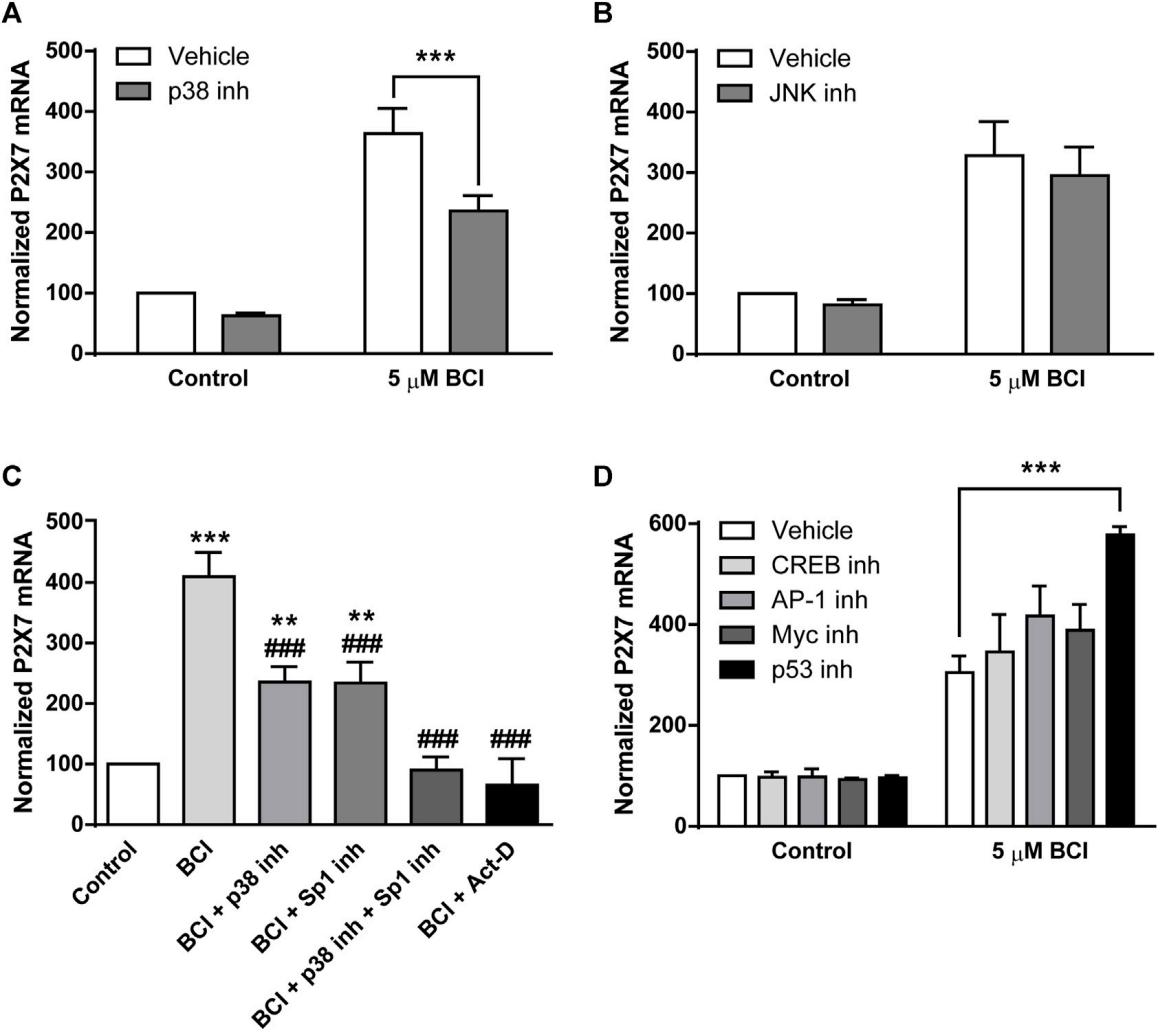
The inhibition of p38 prevents BCI-dependent overexpression of P2RX7 in neuroblastoma cells. N2a cells cultured in SFM were treated with inhibitors of **(A)** p38 (10 μM, SB202190) or **(B)** JNK (10 μM, SP600125) for 10 min before the addition for 24 h of either BCI (5 µM) or the vehicle alone (control). Then, total RNA was extracted from the cells and quantified. The data were normalized to the P2RX7 transcript levels in untreated cells cultured in SFM (set as 100%). The results are the mean ± SEM of six independent experiments in triplicate ***p ≤ .001 vs. vehicle. **(C)** N2a cells cultured in SFM were treated for 10 min with inhibitors of Sp1 (300 nM, mithramycin A), p38 (10 μM, SB202190) or both before adding BCI (5 µM) for 24 h. The transcriptional inhibitor actinomycin D (5 µM) was also assayed. Total RNA was then extracted from the cells and quantified. The data were normalized to the P2RX7 transcript levels in control cells cultured in SFM (set as 100%). The results are the mean ± SEM of five independent experiments performed in triplicate **p ≤ .01, ***p ≤ .001 vs. control; ^###^ *p* ≤ .001 vs. 5 μM BCI. **(D)** N2a cells cultured in SFM were treated for 30 min with inhibitors of CREB (1 μM, 666-15), AP-1 (10 μM, SR11302), Myc (20 μM, KJ Pyr 9) or p53 (20 μM, cyclic pifithrin-α) before the addition of BCI (5 µM) or the either alone (control) for 24 h. Afterwards, total RNA was extracted from the cells and quantified. The data were normalized to the P2RX7 transcript levels in control cells cultured in SFM (set as 100%). The results are shown as the mean ± SEM of six independent experiments performed in triplicate ***p ≤ .001 vs. vehicle.

The data obtained that far demonstrated that BCI produces a strong increase in P2RX7 transcripts and protein in N2a cells, and the inhibition of DUSP1 by BCI appears to be responsible for the increase in p38 and JNK phosphorylation. To further explore the signaling pathway involved in the upregulation of P2RX7 expression induced by BCI, N2a cells in SFM were exposed for 10 min to inhibitors of either p38 (SB202190, 10 µM) or JNK (SP600125, 10 µM) before treating them with BCI (5 µM) for 24 h. Remarkably, inhibition of p38 partially impaired the upregulation of P2RX7 induced by BCI (Figure 7A), whereas JNK inhibition had no effect on P2RX7 transcripts in these cells relative to control cells (Figure 7B). Since SB202190 did not fully prevent the effects of BCI, there would appear to be a mechanism independent of p38 that is also involved in the upregulation of P2RX7 expression induced by inhibiting DUSP1.

Specificity protein 1 (Sp1) is a transcription factor that plays a crucial role in the control of *P2rx7* gene expression in neuroblastoma (Garcia-Huerta et al., 2012). Mithramycin A is an antibiotic that blocks Sp1-dependent gene transcription and when N2a cells in SFM were exposed for 30 min to mithramycin A (300 nM) prior to their treatment for 24 h with BCI (5 µM), there was a partial reduction in the upregulation of P2RX7 induced by BCI (Figure 7C). Hence, Sp1 would also appear to be implicated in the effect of BCI. Most importantly, treating N2a cells with both SB202190 and mithramycin A completely blocked the upregulation of P2RX7 expression induced by BCI. Actinomycin D is an antibiotic that blocks transcription by forming a stable complex with double-stranded DNA. The incubation of N2a cells with actinomycin D (5 µM) totally prevented P2RX7 upregulation, demonstrating that the increase of P2RX7 expression mediated by BCI involves *de novo* synthesis of the transcript.

We previously characterized the promoter sequence of mouse *P2rx7* gene, showing that this 5′-regulatory region not only contains several Sp1 motifs but also, it contains other putative regulatory elements including c-AMP-responsive element binding proteins (CREB), activator protein 1 (AP-1), and Myc/Max heterodimer binding sites (Garcia-Huerta et al., 2012). Importantly, CREB, AP-1 and Myc transcriptional activity can be regulated by various protein kinases including some dependent on p38 (Whitmarsh and Davis, 1996; Deak et al., 1998; Xing et al., 1998; Chen et al., 2006). To assess whether these transcription factors may be implicated in the upregulation of *P2rx7* gene expression induced by BCI, N2a cells in SFM were incubated with specific inhibitors of CREB, AP-1 and Myc transcription factors that potentially bind these regulator elements in the *P2rx7* promoter. We also evaluated the participation of the transcription factor p53, a downstream target of p38. As such, N2a cells in SFM were exposed for 30 min to inhibitors of CREB (666-15, 1 µM), AP-1 (SR11302, 10 µM), Myc (KJ Pyr 9, 20 µM) or p53 (cyclic pifithrin-α, 20 µM) before treating them for 24 h with BCI (5 µM). However, none of the drugs tested diminished the upregulation of P2RX7 expression provoked by BCI (Figure 7D), indicating that the mechanism by which p38 induces *P2rx7* gene transcription is still to be defined.

## 4 Discussion

Neuroblastoma is a tumor of the developing sympathoadrenal lineage that represents the most common solid extracranial tumor of childhood. Patients are often diagnosed under the age of 10 years, and age at diagnosis inversely correlates with the outcome (Cheung and Dyer, 2013; Matthay et al., 2016). To date, the agent that most effectively induces neuroblastoma differentiation is retinoic acid (RA). RA promotes an irreversible reduction in proliferation and an increase in neurite formation in several neuroblastoma cell lines *in vitro* (Seeger et al., 1982). Moreover, the RA derivative, 13-cis RA has become part of the standard of care as it induces differentiation of residual tumor cells after patients receive chemotherapy, surgical resection, and bone marrow transplantation (Matthay et al., 1999). Interestingly, RA-induced neurite outgrowth and neuronal marker expression in neuroblastoma cells is associated with decreases in the expression and activity of P2RX7 (Wu et al., 2009; Orellano et al., 2010). Significantly, N2a differentiation is also induced through the functional inhibition of P2RX7 by selective antagonists or through the siRNA-induced silencing of its expression (Gomez-Villafuertes et al., 2009; Wu et al., 2009). Moreover, P2RX7 activation facilitates the exocytotic release of ATP, which would activate P2RX7 in the same or neighboring neuroblastoma cells, further stimulating its own release and negatively controlling cell differentiation (Gutierrez-Martin et al., 2011). P2RX7 is expressed strongly by primary neuroblastoma cells and cell lines (Raffaghello et al., 2006), and high P2RX7 expression correlates with the poor prognosis of stage IV neuroblastoma patients (Amoroso et al., 2015). Hence, better understanding how the levels of P2RX7 are regulated in neuroblastoma cells may lead to the development of more effective, less toxic therapies. We previously reported that serum deprivation triggers EGFR-dependent activation of the PI3K/Akt pathway in neuroblastoma N2a cells, which is crucial for the Sp1-dependent transcription of *P2rx7* gene (Gomez-Villafuertes et al., 2015). Here, we demonstrated that BCI, an allosteric inhibitor of DUSP1/6 phosphatases, upregulates *P2rx7* gene expression in neuroblastoma cells *via* p38 MAPK, adding to the factors implicated in the control of P2RX7 expression.

DUSP1 and DUSP6 both exhibit tumor suppressor and tumor promoter activity in different cancers, and the same phosphatases may exhibit opposite roles in different tumors (Patterson et al., 2009). For instance, DUSP1 overexpression favors the growth of prostate cancer, driving tumor progression, whereas it facilitates the apoptosis of neuroblastoma cells and therefore has a detrimental effect on tumor cell survival (Shen et al., 2016). By contrast, DUSP6 is upregulated in non-small-cell lung carcinoma but down-regulated in pancreatic cancer, producing tissue-specific suppressor or pro-oncogenic effects (Ahmad et al., 2018). DUSP1 is an inducible nuclear phosphatase that can bind to and dephosphorylate all three major classes of MAPKs (ERK, p38 and JNK) depending on the cell subtype (Raingeaud et al., 1995; Slack et al., 2001). This differs from the activity of DUSP6, a constitutive cytoplasmic phosphatase that binds specifically to ERK1/2 to dephosphorylate and inactivate these MAPKs (Groom et al., 1996; Muda et al., 1996). Significantly, we found that both nuclear DUSP1 and cytosolic DUSP6 phosphatases were expressed strongly in serum deprived N2a neuroblastoma cells. Treating these cells with BCI induced a strong time-dependent upregulation of P2RX7 transcripts, producing an 8-fold increase after 24 h relative to control cells. However, the concentration of BCI was crucial for cell survival, especially after longer treatments (≥24 h), and a small reduction in the BCI concentration from 10 to 5 μM was sufficient to increase P2RX7 transcripts and protein while significantly reducing its unspecific cytotoxicity. Indeed, a recent study demonstrates that BCI can provoke cell death in a dose-dependent manner through a complex mechanism unrelated to the inhibition of DUSP1/6. Unfortunately, the key cytotoxic target of BCI remains to be elucidated, although there is evidence pointing to elements in the mTOR/S6K signaling pathway (Thompson et al., 2022).

DUSPs are phosphatases specialized in the selective dephosphorylation of regulatory Thr and Tyr residues in the distinct MAPKs, and they fulfil important physiological roles as modulators of cell growth, differentiation, and apoptosis provoked by certain extracellular cues (Keshet and Seger, 2010; Cargnello and Roux, 2011; Caunt and Keyse, 2013). The phospho-kinase profile of N2a cells indicated that BCI treatment induced the rapid and transient phosphorylation and activation of both p38 and JNK. Conversely, the phosphorylation of ERK1/2 triggered by serum deprivation in control cells was completely blocked by BCI, an effect that can be explained by the existence of a negative loop between p38 and ERK. Indeed, inhibition of p38 recovered ERK phosphorylation in N2a cells treated with BCI, and BCI enhanced the expression of DUSP1 while it blocked the induction of DUSP6 transcripts mediated by serum deprivation in control cells. Together, these findings point to DUSP1 as a prominent phosphatase involved in the upregulation of P2RX7 induced by BCI. Inhibition of the ERK pathway by p38 can be explained by the activation of different phosphatases, particularly MKPs and type-2 family of protein phosphatases (PP2) (Janssens and Goris, 2001; Junttila et al., 2008). *In vitro* studies indicate that PP2A can dephosphorylate and inactivate both MEK1/2 and ERK1/2 proteins (Sontag et al., 1993). Moreover, transgenic overexpression of a dominant-negative form of the PP2A catalytic subunit in the mouse brain increases MEK1/2 phosphorylation, providing *in vivo* evidence of negative MEK1/2 regulation by PP2A (Kins et al., 2003). Importantly, the involvement of PP2A in p38-mediated MEK1/2 dephosphorylation is supported by the observation that p38 enhances the physical association of endogenous PP2A with the MEK1/2-ERK1/2 complex (Liu and Hofmann, 2004; Grethe and Porn-Ares, 2006). In N2a cells, inhibition of PP2A by okadaic acid prevented the dephosphorylation of ERK induced by BCI, indicating that p38 inhibits the ERK pathway *via* PP2A. Surprisingly, we observed that inhibiting PTEN also prevented the dephosphorylation of ERK induced by BCI in N2a cells. Indeed, a recent study demonstrated that p38 is upregulated by PTEN overexpression and downregulated by PTEN silencing, accompanied by a reduction in ERK1/2 phosphorylation (Zeng et al., 2022). Thus, inhibiting PTEN may downregulate p38 activity, consequently preventing the PP2A-dependent dephosphorylation of ERK1/2.

The signaling pathway involved in the upregulation of P2RX7 expression induced by BCI was also explored here. The increase of P2RX7 expression required *de novo* synthesis of its transcripts since actinomycin D totally abolished the effect of BCI. The p38 inhibitor SB202190 reduced P2RX7 mRNA levels by 50%, indicating that an additional element independent of p38 must be implicated. JNK inhibition did not modify the expression of P2RX7 in control cells or in cells exposed to BCI. We previously identified Sp1 as the main nuclear factor involved in controlling *P2rx7* gene expression in N2a neuroblastoma cells and here, blocking Sp1-dependent transcription with mithramycin A significantly reduced but did not abolish the expression of P2RX7 in BCI-treated cells. Most importantly, exposing N2a cells to both SB202190 and mithramycin A fully impaired the BCI-dependent upregulation of P2RX7 expression, demonstrating that both p38-dependent signaling and Sp1 were involved in this process. The promoter of murine *P2rx7* gene not only comprises Sp1-binding sites but also, other putative regulatory elements like CREB, AP-1 and Myc/Max heterodimer (E-box) binding sites (Garcia-Huerta et al., 2012). Furthermore, the transcriptional activity of CREB, AP-1 and Myc can be regulated by multiple protein kinases, including p38-dependent pathways (Whitmarsh and Davis, 1996; Deak et al., 1998; Xing et al., 1998; Chen et al., 2006). However, inhibiting CREB, AP-1, or Myc dependent transcription did not affect the upregulation of P2RX7 expression induced by BCI in N2a cells, so the transcription factor involved in the p38-dependent upregulation of P2RX7 remains unknown. The additive effect of SB202190 and mithramycin A could indicate that proteins downstream p38 bind and enhance Sp1 activity. For instance, Oct1 interacts with Sp1 and augments its DNA binding affinity by cooperatively binding to regulatory elements and enhance transcription (Strom et al., 1996). Other proteins can bind to Sp1 and activate transcription synergistically. For instance, estrogen receptor proteins bind to Sp1 to increase Sp1-DNA binding to estrogen responsive elements (Porter et al., 1997). Alternatively, transcription factors like AP-2 can superactivate Sp1-dependent transcription by interacting with DNA-bound Sp1 rather than binding directly to DNA (Pena et al., 1999). We also analyzed the effect of p53, a downstream target of p38 that when activated binds to p53-responsive elements in the DNA, thereby initiating transcriptional programs in response to stress signals (Jin and Levine, 2001; Harris and Levine, 2005). Furthermore, p53-dependent expression of the Wip1 protein phosphatase selectively dephosphorylates and inactivates p38 signaling (Takekawa et al., 2000). Here, the inhibition of p53 significantly increased the P2RX7 transcripts in cells treated with BCI, probably due to the stronger phosphorylation and activation of p38. This hypothesis was supported by a decrease in ERK phosphorylation after p53 inhibition in control N2a cells, which can be also explained by a more active p38-PP2A pathway. Alternatively, p53 can interact with Sp1 to negatively regulate transcription at some promoters. For instance, p53 interferes with Sp1 binding to the human telomerase reverse transcriptase gene promoter, preventing its expression and contributing to tumor suppression (Xu et al., 2000). Alternatively, p53 binding to Sp1 can inhibit expression of the cyclin B1 gene by disruption of the recruitment of transcription machinery (Innocente and Lee, 2005).

**FIGURE 8:**
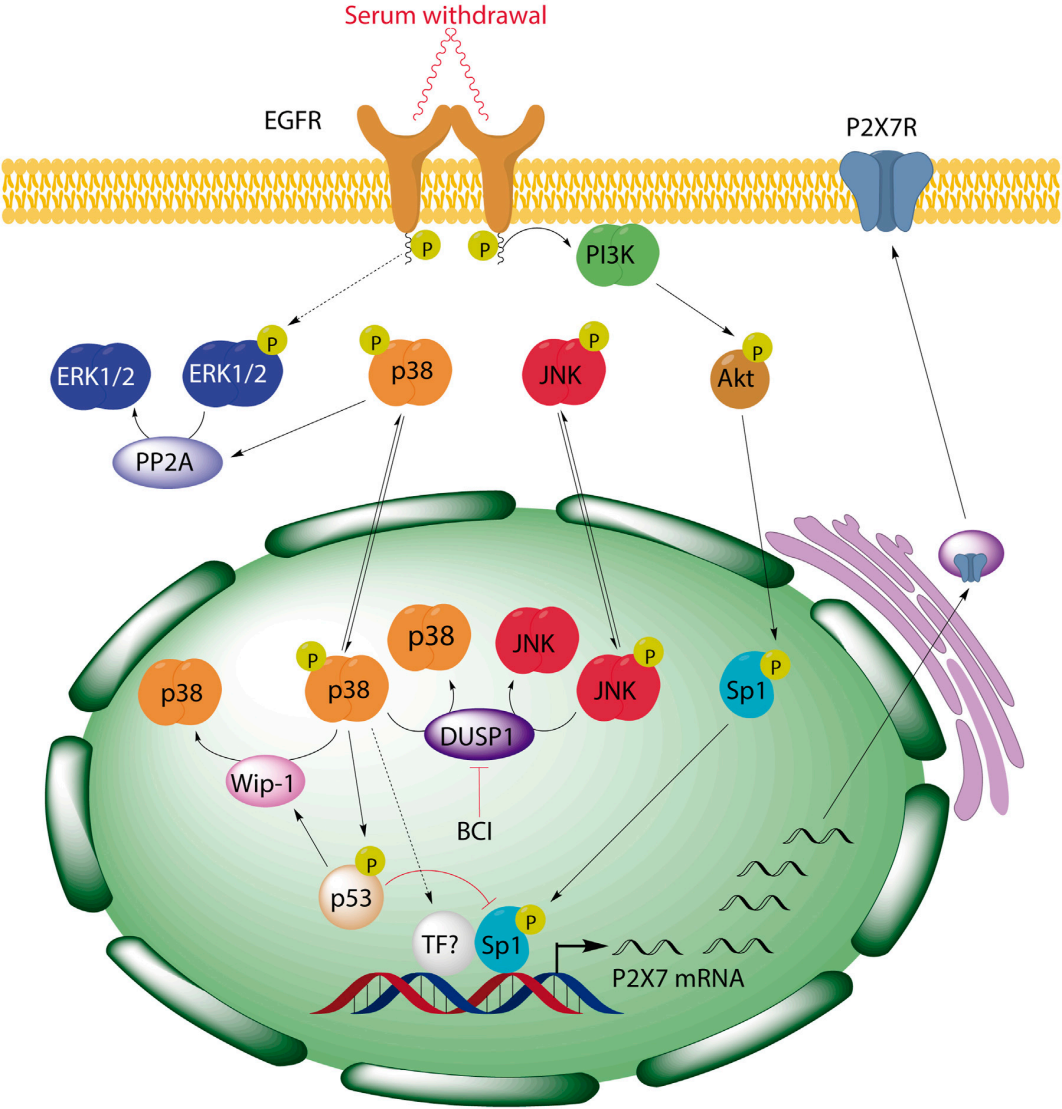
BCI-mediated inhibition of DUSP1 induces the upregulation of P2RX7 through the activation of p38 signaling pathway in neuroblastoma cells. We previously demonstrated that serum starvation triggers EGF-independent activation of EGFR and consequently, activation of PI3K/Akt pathway, resulting in Sp1 phosphorylation and the induction of *P2rx7* gene expression. Here, we have described a negative regulatory loop between p38 and ERK1/2 MAPKs in neuroblastoma cells that involves p38-dependent activation of PP2A and PP2A-dependent dephosphorylation of ERK1/2. Moreover, inhibition of DUSP1 by BCI enhances *P2rx7* gene expression through the activation of p38, which can also be modulated by its downstream target p53, and the participation of Sp1 transcription factor.

In summary, our data highlight DUSP1 as a novel negative regulator of P2RX7 expression in neuroblastoma cells due to the downregulation of the p38 pathway. This negative feedback mechanism could be particularly relevant in tumor microenvironment, where the activation of the p38 may upregulate *P2rx7* gene expression. Based on preclinical studies reporting a strong oncogenic role of this receptor, an increase of P2RX7 facilitates cell proliferation even in scenarios of trophic deprivation, promoting tumor cell proliferation, energy production, migration and invasiveness of cancer cells (Adinolfi et al., 2005; Adinolfi et al., 2012; Qiu et al., 2014; Amoroso et al., 2015; Gomez-Villafuertes et al., 2015; Amoroso et al., 2016). The p38 MAPK pathway can be triggered in response to a plethora of inflammatory cytokines, as well as by pathogens and by environmental stress (Roux and Blenis, 2004; Ashwell, 2006). Moreover, strong p38 activation has been observed in some tumors, promoting cancer cell growth and survival (Greenberg et al., 2002; Gauthier et al., 2005; Junttila et al., 2007). Enhanced p38 activity also correlates with the invasiveness of several cancer cell lines, whereas p38 inhibition reduced their proliferation, survival, and invasion (Johansson et al., 2000; Junttila et al., 2007). Unfortunately, the precise molecular target downstream p38 that drives this effect is yet to be elucidated. Furthermore, we have described a negative regulatory loop between p38 and ERK1/2 MAPKs in neuroblastoma cells that involves p38-dependent activation of PP2A and PP2A-dependent dephosphorylation of ERK1/2 (see simplified scheme in Figure 8). The consequences of ERK dephosphorylation could be also relevant in neuroblastoma, where ERK promotes the activity of various transcription factors such as CREB, Myc, Jun, Fos, Elk-1, Ets, Msk and Atf2. These transcription factors are involved in regulating various cellular physiological processes such as cell proliferation, metabolism, differentiation, cell cycle progression, and metabolism (Muta et al., 2019; Guo et al., 2020). To conclude, our findings have three potential implications for future clinical studies: 1) tumors generated by pathological activation of p38 signaling pathway might also overexpress P2RX7; 2) endogenous alterations or pharmacological inhibition of DUSP1/6 phosphatases might enhance the levels of P2RX7 in neuroblastoma cells, thus facilitating the their survival; and 3) pharmacological interventions targeting the signaling cascade indicated in this study might enhance the effects of combinatorial treatments and reduce their toxicity (e.g., combining selective inhibitors of the p38 pathway, Sp1-dependent transcription and P2RX7).

## Data Availability

The raw data supporting the conclusion of this article will be made available by the authors, without undue reservation.
