# Supplementary material for: BCI, an inhibitor of the DUSP1 and DUSP6 dual specificity phosphatases, enhances P2X7 receptor expression in neuroblastoma cells
Source: Front Cell Dev Biol. 2022 Dec 15;10:1049566. doi: 10.3389/fcell.2022.1049566 (PMC9797830; doi:10.3389/fcell.2022.1049566)
Supplement: Supplementary file 1 [file DataSheet1.docx]

**Supplementary figure 1 BCI upregulates P2RX7 expression in neuroblastoma cells cultured in complete medium.** N2a cells were incubated in complete DMEM médium (10% FBS) or in serum-free medium (0% FBS) for 24 h in the presence of BCI (5 μM) or in its absence (control). Total RNA was extracted from the cells and quantified by Q-PCR. The data were normalized to the P2RX7 transcript levels in control cells cultured in complete medium (set as 100%). The results are the mean ± SEM of three independent experiments performed in duplicate. ****P*≤0.001 *vs* control.

**Supplementary figure 2 BCI upregulates P2RX7 expression in human neuroblastoma cells.** SHSY5Y cells were incubated in SFM for 24 h in the presence of BCI (5 μM) or in its absence (control). Total RNA was then extracted from the cells and quantified. The data were normalized to the P2RX7 transcript levels in cells cultured in control cells, set as 100% (time = 0). The results are the mean ± SEM of two independent experiments performed in triplicate. **P*≤0.05 *vs* control.
